# *Strongylocentrotus intermedius* Extract Suppresses Adiposity by Inhibiting Adipogenesis and Promoting Adipocyte Browning via AMPK Activation in 3T3-L1 Cells

**DOI:** 10.4014/jmb.2404.04041

**Published:** 2024-07-12

**Authors:** Lakshi A. Dayarathne, Jasmadi, Seok-Chun Ko, Mi-Jin Yim, Jeong Min Lee, Ji-Yul Kim, Gun-Woo Oh, Dae-Sung Lee, Won-Kyo Jung, Sei-Jung Lee, Jae-Young Je

**Affiliations:** 1 Department of Food and Nutrition, Pukyong National University, Busan 48513, Republic of Korea; 2 National Research and Innovation Agency, Research Center for Food Technology and Processing, Gunungkidul, 55861, Indonesia; 3 National Marine Biodiversity of Korea (MABIK), Seochun 33662, Republic of Korea; 4 Major of Biomedical Engineering, Division of Smart Healthcare, Pukyong National University, Busan 48513, Republic of Korea; 5 Major of Human Bioconvergence, Division of Smart Healthcare, Pukyong National University, Busan 48513, Republic of Korea

**Keywords:** *Strongylocentrotus intermedius*, lipogenesis, lipolysis, adipocyte browning, AMPK

## Abstract

The current study aimed to determine whether *Strongylocentrotus intermedius* (*S. intermedius*) extract (SIE) exerts anti-obesity potentials employing 3T3-L1 cells as in vitro model. Herein we reported that treatment of SIE for 6 days reduced lipid accretion and triglyceride content whereas it increased the release of free glycerol. The inhibited lipid accumulation and induced lipolysis were evidenced by the downregulation of lipogenesis proteins, such as fatty acid synthase and lipoprotein lipase, and the upregulation of hormone-sensitive lipase expression. Furthermore, the downregulation of adipogenic transcription factors, including peroxisome proliferator-activated receptor gamma, CCAAT/enhancer-binding protein α, and sterol regulatory element-binding protein 1, highlights that reduced lipid accumulation is supported by lowering adipocyte differentiation. Additionally, treatment activates brown adipocyte phenotype in 3T3-L1 cells by inducing expression of brown adipose tissue-specific proteins, such as uncoupling protein 1 and peroxisome proliferator-activated receptor-γ coactivator 1α. Moreover, SIE induced the phosphorylation of AMP-activated protein kinase (AMPK). The pharmacological approach using AMPK inhibitor revealed that the restraining effect of SIE on adipogenesis and promotion of adipocyte browning were blocked. In GC-MS analysis, SIE was mainly composed of cholest-5-en-3-ol (36.71%) along with saturated and unsaturated fatty acids which have favorable anti-obesity potentials. These results reveal that SIE has the possibility as a lipid-lowering agent for the intervention of obesity.

## Introduction

Obesity develops due to a positive energy balance between energy intake and expenditure, ultimately resulting in the pathological expansion of adipocytes [1]. The process of cellular differentiation that converts pre-adipocytes to mature adipocytes is called adipogenesis and involves the activation of many transcription factors, including CCAAT/enhancer-binding protein alpha (C/EBPα), peroxisome proliferator-activated receptor gamma (PPARγ), and sterol regulatory element-binding protein 1 (SREBP-1) [2]. These transcription factors regulate lipid homeostasis via modulating gene expression profiles related to fat accumulation, fatty acid transport, and lipolysis [3]. Maintaining an appropriate energy balance is important for treating obesity. Many of the anti-obesity drugs approved to date suppress energy intake by reducing fat absorption or suppressing appetite, but these drugs have serious drawbacks [4]. Thus, alternative approaches for improving energy consumption are crucial to combat obesity complications.

Mammalian adipose tissue is categorized into two main types: white adipose tissue (WAT) and brown adipose tissue (BAT). WAT accumulates chemical energy in the form of triglyceride (TG) and contributes to weight gain. On the other hand, BAT dissipates chemical energy as heat and enhances energy utilization via the activation of uncoupling protein 1 (UCP-1) [5]. The recent findings in obesity research demonstrate that sustained cold exposure and adrenergic stimulation can trigger browning which converts WAT to brown-like (beige) adipocytes in WAT, representing an inducible form of BAT [6]. These beige adipocytes increases energy expenditure upon their formation, which also serves a vital function in energy regulation. [7]. Thus, induction of browning in WAT has become an attractive therapeutic concern for diet-induced obesity [8]. Numerous clinical studies have focused on targeting beige adipocyte formation for the treatment of obesity. However, many compounds, such as catecholamines, have been discontinued due to side effects, including detrimental effects on the heart, bones, and autonomic nervous system [9, 10]. Many dietary agents and natural compounds have been known to recruit beige adipocytes in WAT and decrease fat accumulation in mammals [11, 12]. Therefore, to achieve safety and multifunctionality, an increasing focus on natural products that can induce adipocyte browning has become a research concern.

Because of the peculiarities in living conditions, such as specific dietary preferences and high-pressure habitats, marine organisms are enriched with biologically active substances and ingredients of functional foods [13]. The gray sea urchin *Strongylocentrotus intermedius* (*S. intermedius*) a highly valued seafood belonging to the family Strongylocentrotus is naturally distributed in shallow coastal zones of the East Sea of Korea. *S. intermedius* is rich in various phenols and other bioactive compounds that exhibit bactericidal, hypotonic, and anti-allergic activities. Furthermore, gray sea urchin have long been used in the Asian-Pacific region as a remedy to improve general living conditions and treat several diseases [14-16]. However, as per our current knowledge, the anti-obesity potentials of *S. intermedius* have not yet been explored. Thus, the objective of this study is to investigate whether *S. intermedius* has an anti-obesity effect and to provide evidence that it inhibits adipogenesis and stimulates adipocyte browning in 3T3-L1 preadipocytes.

## Materials and Methods

3T3-L1 cells were obtained from the American Type Culture Collection (ATCC, USA). Additional reagents, all of the analytical grade, were obtained from Sigma-Aldrich (USA). Cell culture reagents were purchased from Life Technologies (Gibco BRL, USA). All anti-bodies were products from Santa Cruz Biotechnology (USA).

### Cell Culture

Cells were grown in Dulbecco's modified Eagle's medium (DMEM) supplemented with 10% bovine calf serum; 1% penicillin/streptomycin (PS) in a humidified atmosphere containing 5% CO_2_ at 37°C.

### Preparation of *Strongylocentrotus intermedius* Extract

The *S. intermedius* extract (SIE) was generously provided by the National Marine Biodiversity Institute of Korea (MABIK). *S. intermedius* was collected from Goseong, Gangwon Province, Republic of Korea, and a voucher specimen (NP-0224) was placed at MABIK. After washing *S. intermedius* with running water it was stored at -80°C and subsequently subjected to lyophilization and homogenization using a grinder. Then the resulting powder was wrenched out with 70% (v/v) EtOH (1:10w/v) for 1h (five repeats) by sonication. The dried extract was obtained by vacuo evaporation and stored at −80°C till the biological effect assessment.

### 3T3-L1 Cells Differentiation

**Fig. 1:**
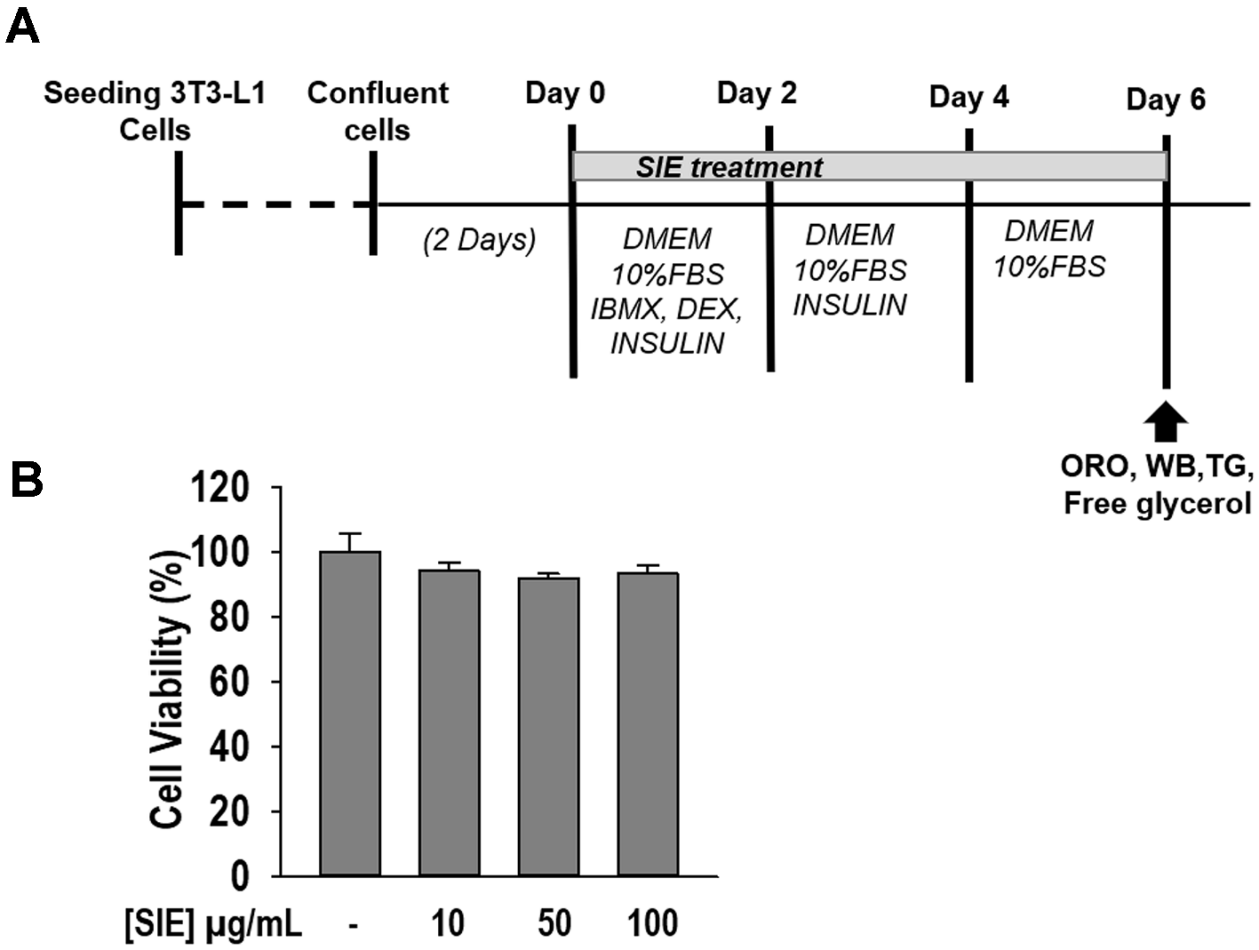
Cell viability of SIE in 3T3-L1. Differentiation procedure and assay schedule (**A**) 3T3-L1 cell viability (**B**) The cells were treated with 10-100 μg/ml of SIE for 48 h, and cell viability was measured by MTT assay. All values given are means ± SD (*n* = 3).

Post confluence 3T3-L1 cells were incubated with the differentiation initiation media (MDI) containing DMEM, 1% PS, 10% fetal bovine serum (FBS), 3-isobutyl-1-methylxanthine (IBMX), dexamethasone (DEX), and insulin for 48 h. Subsequently, the MDI was replaced with post-differentiation media (DMEM, 1% PS, 10% FBS, and insulin) for another 48 h. After 48 h of incubation, media were replaced with normal growth media every other day until complete differentiation of adipocytes. To observe the effects of SIE on adipogenesis, cells were cultured in MDI with or without SIE until differentiation. After 6 days, matured adipocytes were used for proceeding experiments (Fig. 1A).

### 3-(4,5-Dimethylthiazol-2-yl)-2,5-Diphenyltetrazolium (MTT) Assay

Cellular toxicities of SIE in 3T3-L1 cells were observed by performing MTT assay. Confluent cells were treated with different concentrations (0~100 μg/ml) of SIE for 48 h, followed by incubation with MTT working reagent for 3 h. Formazan production, indicating cell viability was quantified by a microplate reader (Tecan, Switzerland).

### Oil Red O Staining

Lipid accumulation in 3T3-L1 cells was observed using Oil red O staining. After fixing cells with 10% formalin for 1 h, freshly prepared Oil Red O working solution (O0625 Sigma-Aldrich, USA), was added and allowed to stain for 10 min. The unbound stain was washed off with distilled water. An inverted microscope (DMI6000, Leica, Germany) was used to capture the images. The stain was then extracted in isopropanol for quantification analysis.

### TG Assay

Cellular TG content was quantified by a commercial colorimetric TG assay kit (Biomax, Republic of Korea) following the manufacturer’s instructions.

### Free Glycerol Release Assay

Free glycerol release into the cultured medium by mature adipocytes was assessed by free glycerol reagent (F6428 Sigma-Aldrich) following the manufacturer’s instructions.

### Western Blot Analysis

Matured adipocytes were lysed with ice-cold RIPA buffer with a protease inhibitor mixture. The whole-cell lysates were centrifuged and the supernatant was collected. Equal amounts of protein, determined by BCA assay were mixed with loading buffer, followed by gel electrophoresis, and incubated with anti-bodies. A chemiluminescence ECL assay kit (BWP0200, WestGlow PICO PLUS, Biomax, Republic of Korea) was used to visualize the bands, and images were taken using a Davinch-Chemi Imager (CAS400SM, Core Bio, Republic of Kore). Densitometry analysis was performed by ImageJ analysis software.

### Total Flavonoid Content (TFC) and Total Phenol Content (TPC)

TFC in SIE was determined according to a previously reported method [17]. Briefly, SIE was mixed with 5%NaNO_2_ and 10% AlCl_3_ followed by incubation of 50 min. Subsequently, the absorbance was obtained at 510 nm. Rutin solution was used as the reference compound. TFC was expressed as mg rutin equivalents per gram of dried extract (mg RE/g).

TPC present in SIE was estimated by using the Folin–Ciocalteu method described earlier [18]. Briefly, the desired amount of extract was mixed with Folin-Ciocalteu solution and incubated for 2 h, after which the absorbance was recorded at 600 nm. Gallic acid was used as the standard reference. The amount of TPC was denoted as mg gallic acid equivalents per gram of dried extract (mg GAE/g).

### Identification of the Chemical Composition of SIE

For the characterization of the chemical composition of SIE, GC-MS analysis was performed. Two microliters of sample dissolved in methanol were injected into a Shimadzu QP-2010 Ultra GC-MS with an Agilent DB-5MS UI column (30 m × 0.25 mm × 0.25 μm). The active compounds were separated using helium as the carrier gas at a rate of 1.0 ml/min. The splitless mode injection (split ratio of 50:1) was carried out, and the injection temperature was adjusted to 280°C. The oven temperature was initially set at 60°C, held for 2 min followed by injection, then increased to 200°C (rate of 10°C/min), and afterwards raised to 320°C at a ramp rate of 5°C/min and held for 20 min. The ion source and interface temperature were set at 200°C and 250°C, respectively. Under electron impact ionization at 70 eV of electron energy, mass spectra were recorded within the scanning range of m/z 40–600. The spectra of the unknown constituents of the *S. intermedius* fraction obtained were compared with the authentic standard mass spectra of known constituents stored in the National Institute Standard and Technology (NIST), NIST V 11 data library.

### Statistical Analysis

Statistical analysis was performed using one-way analysis of variance (ANOVA) and Duncan’s test using Sigma Plot 12.0 (Systat Software Inc., USA). Normality and homogeneity of variables were assessed using the Shapiro-Wilk test and Levene Median test, respectively. Results were means±SD (*n*=3) and *p* < 0.05 denotes statistically significant differences among the groups.

## Results

### Cell Cytotoxicity of SIE

To assess the cytotoxic effect of SIE on 3T3-L1 cells, MTT assay was performed. The results are shown in Fig. 1B. SIE did not show any significant cell damage up to 100 μg/ml concentration. Thus, proceeding experiments were conducted at non-toxic concentrations of SIE.

### Anti-Adipogenic Effect of SIE

**Fig. 2:**
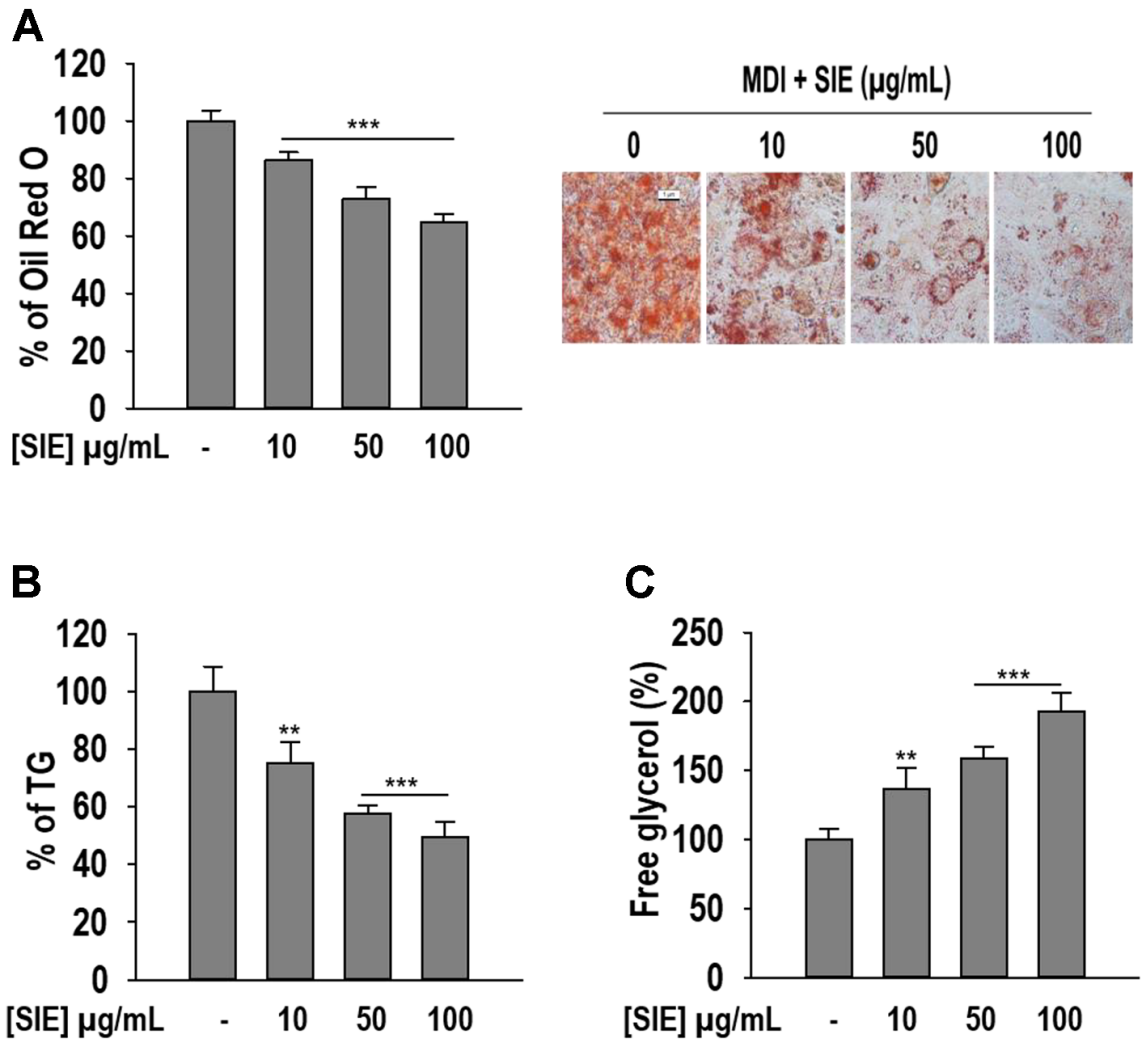
Lipid accumulation, TG content, and free glycerol release by SIE in 3T3-L1 cells. (**A**) Oil Red O images and quantitative analysis of lipid accumulation; (**B**) TG accumulation; (**C**) free glycerol release. Cells were differentiated in the presence and absence of extract for 6 days as described in material and methods followed by staining with Oil Red O reagent, colorimetric determination of TG content, and free glycerol release assay. All data given are means ± SD (*n* = 3). **p < 0.01 and ***p < 0.001.

To observe the effect of SIE on lipid accumulation, 3T3-L1 cells were differentiated in the presence of SIE for 6 days. Morphological changes in adipocytes due to lipid accumulation were observed by Oil Red O staining. As depicted in Fig. 2A, adipocytes without SIE treatment tend to accumulate more lipids compared to adipocytes with SIE. Treatment with SIE significantly reduced (*p* < 0.001) lipid accumulation, in which 100 μg/ml of SIE reduced lipid accumulation approximately by 60%. To further confirm the effect of SIE on adipogenesis, TG accumulation was quantified. A notable increase in TG accumulation was observed in the absence of SIE (control adipocyte), in contrast, SIE treated adipocytes remarkably reduced the intracellular TG content. SIE at 100 μg/ml reduced TG levels by 50%. To observe whether the anti-adipogenesis effect of SIE is associated with lipolysis, lipolytic ability of SIE was observed by quantifying free glycerol release to the medium. The results are illustrated in Fig. 2C. Treatment with SIE augmented the spontaneous release of free glycerol with increasing concentrations. The secreted free glycerol was nearly 130%, 160%, and 190% at the concentrations of 10, 50, and 100 μg/ml, respectively. In brief, the above results reveal that SIE suppresses adipogenesis and induces lipolysis in 3T3-L1 adipocytes.

### SIE Effect on Adipocyte Protein Expressions

**Fig. 3:**
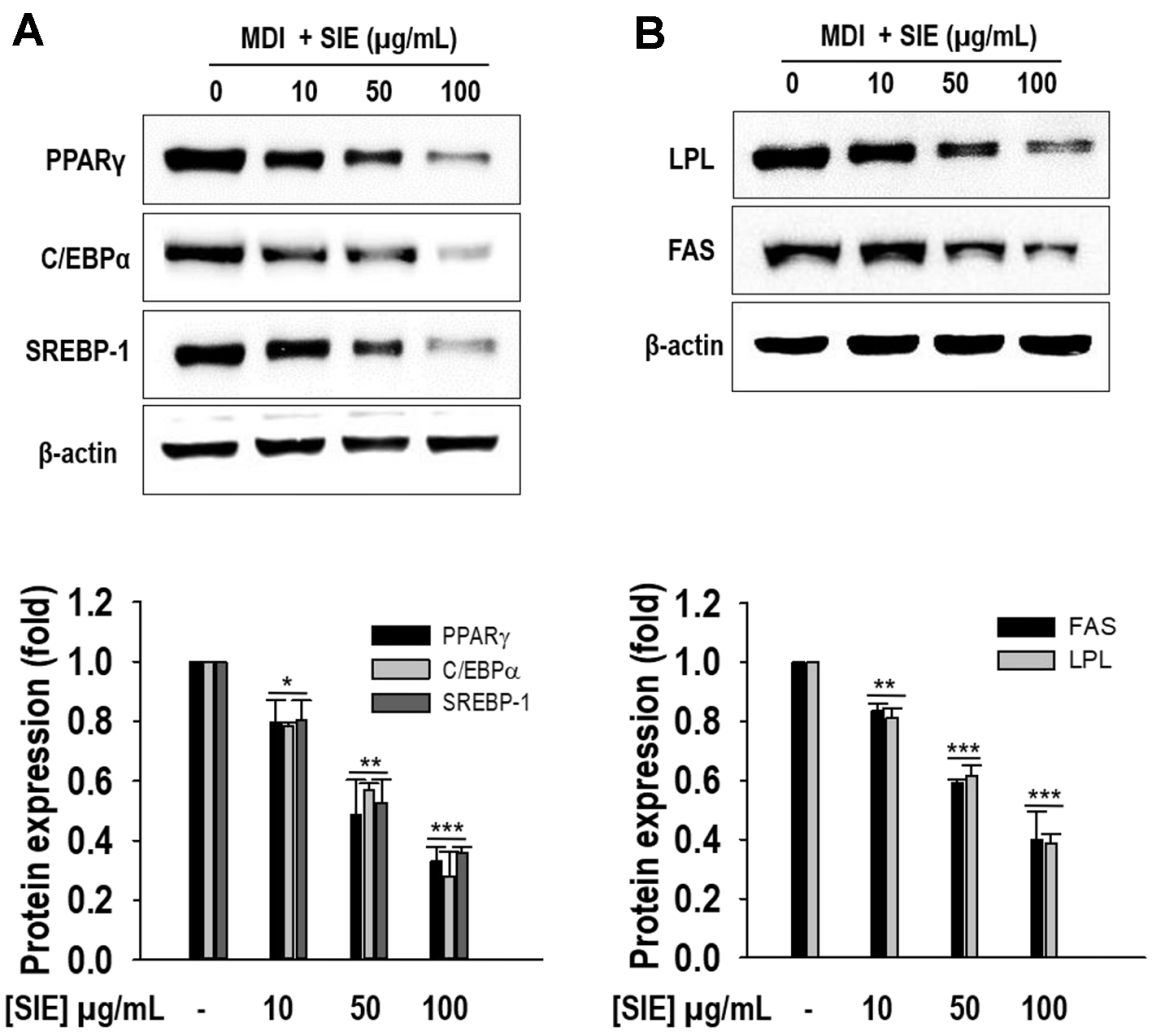
Expressions of adipogenesis and lipogenesis proteins by SIE in 3T3-L1 cells. (**A**) Protein expressions of PPARγ, C/EBPα, and SREBP-1; results are expressed as fold changes compared to the band of β-actin; (**B**) Protein expressions of FAS and LPL; results are expressed as fold changes to the band β-actin. Cells were differentiated in the presence and absence of extract for 6 days as described in materials and methods followed by Western blot analysis. All data given are means ± SD (*n* = 3). *p < 0.05, **p < 0.01, and ***p < 0.001.

Western blot analysis was conducted to examine whether SIE affected protein expression of adipocyte-specific transcription factors. Compared to the differentiated control adipocytes (untreated adipocytes), adipocytes treated with SIE showed significantly (*p* < 0.05) reduced protein expression of PPARγ, C/EBPα, and SREBP-1 (Fig. 3A). 100 μg/ml of SIE reduced the protein expressions by 0.3-fold, 0.28-fold, and 0.35-fold for PPARγ, C/EBPα, and SREBP1, respectively. This study further examined whether SIE regulates expressions of adipogenic target proteins. As shown in Fig. 3B, the untreated adipocytes increased expression of fatty acid synthase (FAS) and lipoprotein lipase (LPL). However, SIE treatment dose-dependently downregulated the expression of FAS and LPL. The results unveiled that SIE modulates adipogenesis by taking over the control of adipogenesis-related key proteins.

### Stimulation of AMPK and Expression of Lipolytic and Brown Adipocyte-Specific Proteins by SIE

**Fig. 4:**
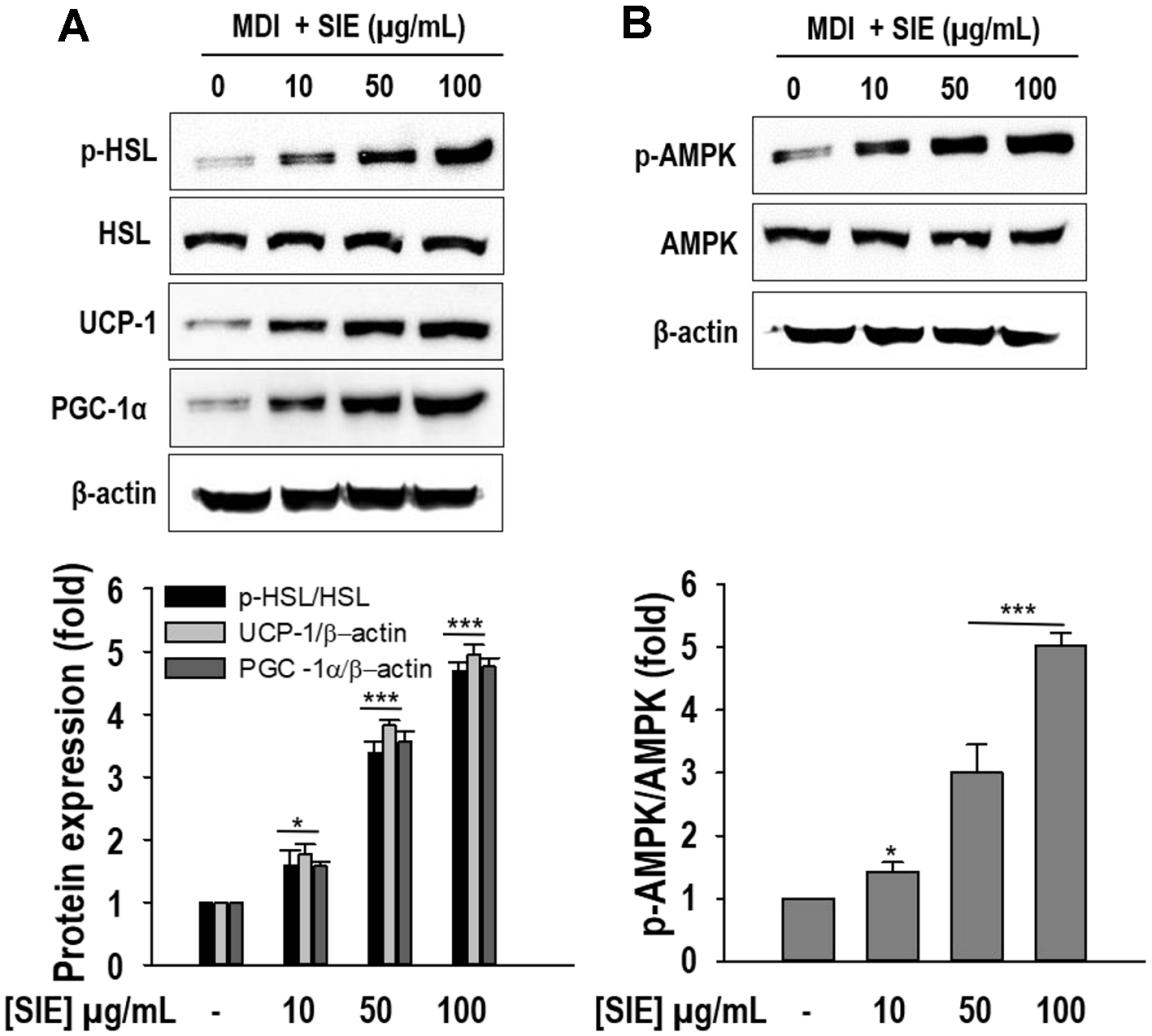
Expression of AMPK, HSL, PGC-1α, and UCP-1 by SIE in 3T3-L1 cells. (**A**) Protein expressions of p-HSL, PGC-1α, and UCP-1; results are expressed as fold changes compared to the band of HSL and β-actin. (**B**) Protein expressions of p-AMPK; results are expressed as fold changes compared to the band of AMPK. Cells were differentiated in the presence and absence of extract for 6 days as described in materials and methods followed by Western blot analysis. All data given are means ± SD (*n* = 3). *p < 0.05 and ***p < 0.001.

Since SIE treatment reduced TG accumulation and increased free glycerol release, further confirmation of its effect on lipolysis was sought by observing phosphorylation of HSL by performing western blotting. As depicted in Fig. 4A, phosphorylation levels of HSL were significantly (*p* < 0.05) increased with SIE treatment, whereas the untreated adipocytes showed weak phosphorylation levels of HSL. Notably a 4.6-fold increase compared to the untreated adipocytes was observed at the highest concentration of SIE.

To evaluate the browning effect of SIE, cells were differentiated along with different concentrations of SIE for 6 days. Subsequently, expression of brown fat markers UCP-1 and PGC-1α was observed via western blotting. As expected, low levels of UCP-1 and PGC-1α expression were detected in the untreated adipocytes. In contrast, SIE treatment considerably induced the expression of UCP-1 and PGC-1α and the induction was dose-dependent (Fig. 4A). Furthermore, this study investigated whether SIE influences lipid metabolism and browning through AMPK activation in 3T3-L1 preadipocytes. SIE treatment substantially (*p* < 0.05) increased the phosphorylation of AMPK (p-AMPK) and 100 μg/ml of SIE resulted in a 5-fold up-regulation of p-AMPK compared to the untreated adipocytes (Fig. 4B). The result suggests that increased lipid metabolism and browning effect of SIE might be attributed to AMPK activation in adipocytes.

### SIE Shows Anti-Adipogenesis Effect through Phosphorylation of AMPK

**Fig. 5:**
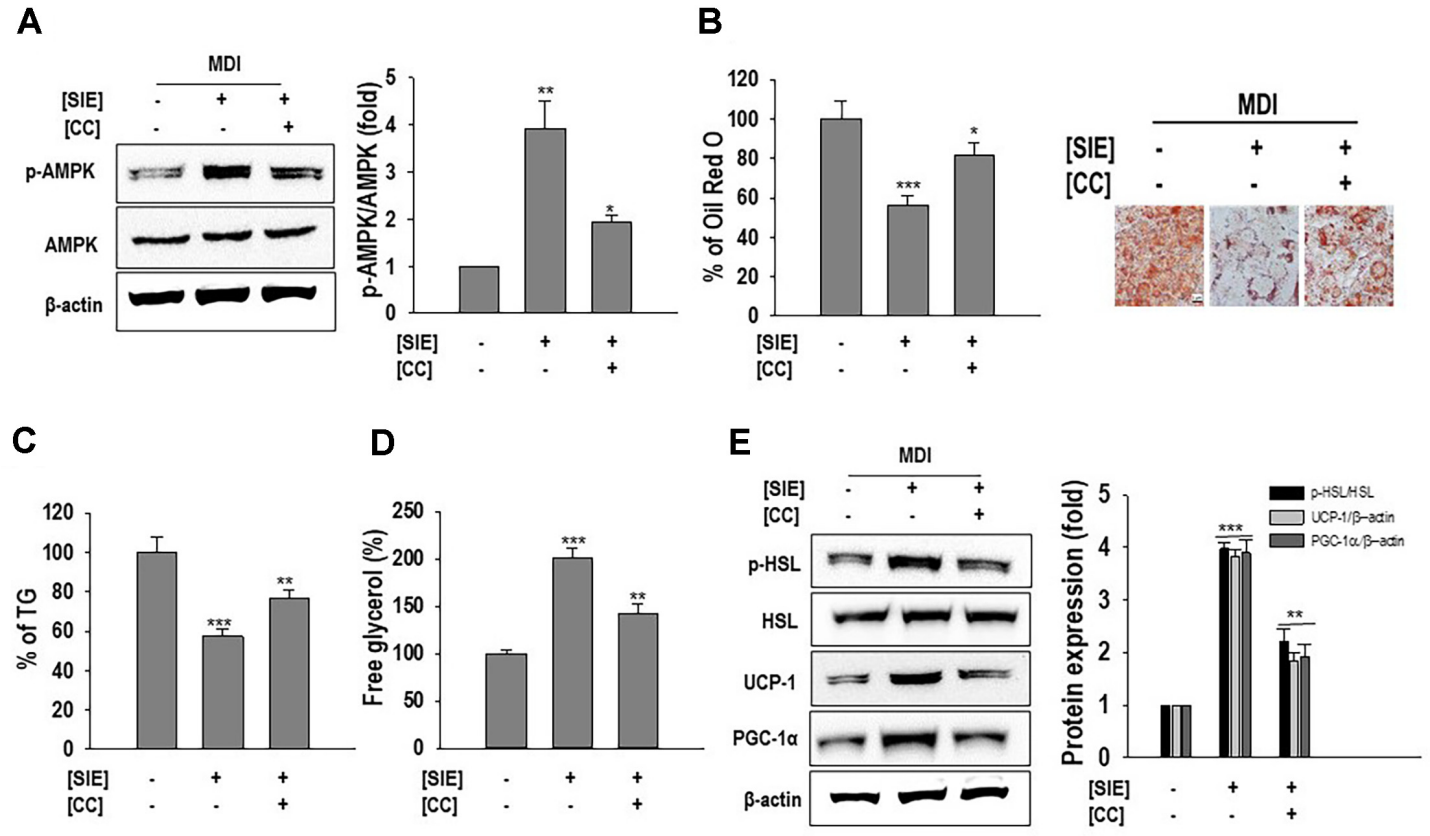
AMPK inhibitor attenuates adipogenesis inhibition by SIE. (**A**) Protein expression of p-AMPK. (**B**) Representative images and quantitative analysis of lipid accumulation; (**C**) TG accumulation; (**D**) Free glycerol release; (**E**) Protein expression of p-HSL, PGC-1α, and UCP-1; results are expressed as fold changes compared to the band of HSL and β- actin respectively. Cells were differentiated and treated with extract as described in materials and methods with 2 h pretreatment of Compound C (**CC**) (10 μM) followed by Western blot analysis, Oil Red O staining, TG assay, and free glycerol release. All data given are means ± SD (*n* = 3). *p < 0.05, **p < 0.01, and ***p < 0.001.

To further validate the role of AMPK in inhibiting lipogenesis and inducing browning in 3T3-L1 preadipocytes, its activity was specifically inhibited by AMPK antagonist (Compound C/Dorsomorphin), and results are presented in Fig. 5. As anticipated, increased level of p-AMPK by SIE was significantly reduced with compound C treatment. Moreover, the reduced lipid accumulation and TG levels by SIE were increased by pretreatment with compound C, whereas the increased free glycerol release was reduced (Fig. 5B-5D). More importantly, the elevated protein levels of UCP-1 and PGC-1α were also suppressed with compound C pretreatment (Fig. 5E). These results confirmed that SIE induced the browning and improved lipid metabolism via p-AMPK signaling pathway in 3T3-L1 adipocytes.

### Chemical Constituents of SIE

TPC and TFC of SIE amounted to 17.78 ± 0.08 mg GAE/g and 12.67 ± 0.1 mg RE/g, respectively. The major active compounds responsible for anti-adipogenic potential of SIE were identified through GC-MS analysis. The GC-MS spectrum of SIE acquired the existence of 12 major constituents with different retention times (RT). These constituents were recognized through mass spectrometry and compared with NIST library. Table 1 summarizes the constituents of SIE. Based on the peak areas, cholest-5-en-3-ol (36.71%), n-hexadecanoic acid (17.76%), 1,2-benzenedicarboxylic acid, dioctyl ester (11.93%), tetradecanoic acid (4.99%) and were found to be the major compounds. Furthermore, the presence of other fatty acids including 9-octadecenoic acid and 9-hexadecenoic acid was also confirmed by GC-MS analysis.

## Discussion

Effective approaches to obesity management include inhibiting adipocyte differentiation, lipogenesis, and stimulating lipolysis [19]. In addition, trans-differentiation of WAT to BAT-like adipocytes through increased energy expenditure has garnered significant attention [20]. In particular, plenty of studies have highlighted the potential anti-obesity activity of marine sources, particularly in influencing the expression of adipogenic and thermogenic proteins. [21-24]. However, the marine environment being one of the largest biodiversity pools remains underexplored. Therefore, as part of our ongoing research on searching marine sources with anti-obesity activity, for the first time, the present study firmly established anti-adipogenesis potential of SIE in in vitro context.

Adipose-specific proteins, including transcription factors PPARγ, C/EBPα, and SREBP-1, have significant roles in lipid accumulation and regulation of adipocyte differentiation, specifically by taking over the control of their subsequent genes [25]. PPARγ and C/EBPα are recognized as major transcription factors that stimulate transcriptional activation in adipocytes [26]. SREBP-1 is associated with adipocyte differentiation and cholesterol synthesis [27]. Previous studies reported that SREBP-1 regulates adipogenic proteins including FAS and LPL, the key proteins involved in fatty acid metabolism, thereby affecting adipogenesis [28]. Thus, suppressing the adipogenic protein expression is an effective approach for inhibiting adipocyte differentiation and lipid accumulation. Accordingly, we differentiated the adipocytes in the presence of SIE and found that SIE effectively reduced lipid accumulation, and TG levels, while also reducing expressions of PPARγ, C/EBPα, SREBP-1, FAS, and LPL.

Next, we examined whether SIE could induce lipolysis during adipocyte differentiation in 3T3-L1 preadipocytes, as expected SIE treatment significantly increases free glycerol release and elevated p-HSL levels. Lipolysis is a major metabolic process that hydrolyses TG to facilitate the body's energy demand. Of note, lipolysis stimulation results in a decrease in fat accumulation in adipocytes, rendering it a promising pharmacological target [3]. Adipocyte lipolysis is mainly regulated by HSL activity, which acts as the rate-limiting enzyme that hydrolyzes diglycerides and plays a role in mobilizing TG from adipocytes [29]. Therefore, activation of HSL could be considered as a strategic approach for adipogenesis treatment.

Finally, SIE's ability to induce adipocyte browning was observed. Several studies have discovered that inducing adipocyte browning effectively improves the body’s energy metabolism and ameliorates insulin resistance in obesity conditions, leading to weight loss [30, 31]. PGC-1α, a hallmark regulator in adipocyte browning has an important role in mitochondrial biogenesis and promotes adipocyte browning by activating several adipocyte browning-related genes specifically UCP-1 [32]. Typically, beige adipocytes exhibit high expression of UCP-1, a transmembrane protein located in the inner mitochondrial membrane. It is involved in adaptive thermogenesis in which heat is generated instead of ATP by disrupting the proton gradient of the membrane which uncouples substrate oxidation in oxidative phosphorylation [33]. In the present study, we found that SIE has significantly up-regulated the expression of adipocyte browning-specific proteins PGC-1α and UCP-1, implying that its anti-adipogenesis effect was supported by adipocyte browning.

Previous studies reported that activation of AMPK promotes beige adipocyte development through expression of PGC-1α and UCP-1 [34-36]. Furthermore, AMPK activation inhibits adipogenesis by reducing lipid biosynthesis, promoting lipolysis, and inhibiting adipocyte differentiation [37, 38]. AMPK is a heterotrimeric enzyme, activated by increased AMP: ATP ratio during metabolic stress [39]. Phosphorylated AMPK then triggers catabolic pathways such as fatty acid oxidation and deactivates anabolic pathways including lipid synthesis and storage, consequently proposing AMPK as a probable pharmacological target for obesity treatment [37, 40]. Therefore, to further elaborate on the mechanism for the anti-adipogenesis effect of SIE, we observe phosphorylation of AMPK. In this study, SIE treatment significantly increases the levels of p-AMPK. Furthermore, exposure to AMPK inhibitor (compound C) reversed the observed anti-adipogenic effects of SIE, reducing lipid accumulation and TG levels and increasing free glycerol release. In addition, the reduced expression of beige adipocyte markers upon AMPK inhibition strengthened our hypothesis that the anti-adipogenic effects of SIE are mediated by AMPK activation.

To observe the chemical compounds which is responsible for favorable anti-obesity effects of SIE, GC-MS analysis was performed. SIE contained several fatty acids, esters, and sterols in abundance. Lipids, particularly, fatty acid content, are widely recognized as the key component that predicts the nutritional worth of seafood with numerous studies documenting their essentiality for human health [41, 42]. A previous study on lipidomic profiling of *S. intermedius* found high amounts of saturated and unsaturated fatty acids mainly polyunsaturated fatty acids (PUFA) [43]. Similarly, SIE used in the present study was rich in several saturated fatty acids namely tetradecanoic acid, pentadecanoic acid, n-hexadecenoic acid, and unsaturated fatty acids including arachidonic acid, 9-hexadecanoic acid, 9-octadecanoic acid, cis-5,8,11,14,17-eicosapentaenoic acid, and erucic acid. Tetradecanoic acid boosts HDL-cholesterol levels and exhibits preventive effects on atherosclerosis, when orally administered, improves glucose tolerance while reducing body weight gain [44, 45]. n-hexadecenoic acid (palmitic acid-PA), a saturated fatty acid commonly present in animal and human tissues. Some studies extensively document the detrimental impact of palmitic acid such as lipotoxicity. Nevertheless, the majority of these findings are based on in vitro cell cultures and in vivo obese models which used exceedingly elevated levels of dietary PA as a singular fatty acid. This overlooks the fact that such conditions are untenable for humans, as dietary PA fails to change its tissue concentration [46]. Therefore, with the above mentioned we believed that dietary extract rich with palmitic acid would be beneficial for obesity treatment. In line with this conjecture, a study on palmitic acid-rich green algal extract (*Caulerpa lentillifera*) was reported for reduced lipid accumulation and TG content in high glucose-fed worms suggesting a potential food supplement for obesity treatment [47]. Furthermore, our previous work on brown algal extract (*Dictyopteris divaricata*) which contained palmitic acid (11.46%) has shown an anti-adipogenesis effect by modulating adipocyte differentiation and inducing lipolysis in 3T3-L1 cells [48]. Salman *et al*. show that the combination of 9-octadecenoic acid (oleic acid) treatment with exercise has demonstrated remarkable efficacy in combating obesity while promoting beige adipocyte formation in obese rats [49]. SIE used in the study contained significant amounts of 1,2-benzenedicarboxylic acid, dioctyl ester which might contribute to its anti-adipogenesis effect. A previous study on water clover (*Marsilea crenata* C. Presl.) identified 1,2-benzenedicarboxylic acid as a potent inhibitor of HMG-CoA reductase, a key enzyme in cholesterol synthesis downstream of SRBP-1 [50].

Sterols from marine sources have been shown to reduce low-density lipoprotein (LDL) cholesterols levels [21]. As reported previously, cholesterol was the major sterol available in most marine crustacean extracts [51]. Based on GC-MS analysis, 36.71% of the extract was cholest-5-en-3-ol generally referred as cholesterols, an essential metabolite vital for membrane structure and as a precursor of bile acids, and steroid hormones [52]. However, studies have opened up the fact that high cholesterols intake is linked with cardiovascular risks. Although dietary intake of cholesterols has increased total serum levels previously [53], research suggests that consumption cholesterol-rich foods like sea urchin does not necessarily elevate plasma or hepatic total cholesterols [54, 55]. Similarly, another study reported that egg consumption, another well-aware dietary cholesterol source, did not alter the human's serum cholesterol levels [56]. Thus, sea urchins rich in cholesterols are of health-safe properties, suggesting SIE as an effective functional food for obesity treatment. Although previous studies on anti-adipogenesis effects of *S. intermedius* are lacking, other research supports sea urchin intake is beneficial for obesity treatment. For instance, Yamamoto *et al*. reported that sea urchin (*Mesocentrotus nudus*) intake led to decreased body, liver, and visceral fat mass and reduced TG levels in mice. In addition, it induced UCP-1 in BAT, thereby exerting protective effects against lipid accumulation in high-fat diet-fed mice [54]. Taking these facts into consideration, we propose that SIE utilized in the study possesses a rich array of chemicals with anti-obesity properties.

Although this study reports the anti-obesity effect of SIE, it does possess certain limitations. The chemical profiling of SIE requires further refinement through isolation and purification to identify the compounds with promising anti-obesity properties. In addition, herein the results provided preliminary data indicating possible anti-obesity effects of SIE in in vitro, further research using in vivo obese models is essential to elaborate these research outcomes.

## Conclusion

The present findings provided evidence that SIE exerts a multi-modulatory function to inhibit the progression of adipogenesis in 3T3-L1 cells. SIE reduces lipid accumulation by down-regulating pro-adipogenic and lipogenic proteins with increased lipid metabolism. Moreover, SIE exhibits additive anti-adipogenic effects by stimulating lipolysis and adipocyte browning while promoting the expression of BAT-specific markers and this was mediated by AMPK activation. Consequently, these findings propose SIE as a promising therapeutic option that could be utilized as a dietary intervention for obesity management.

## Figures and Tables

**Table 1:** Chemical composition of SIE.

R. time	Compound name	Molecular formula	Peak area (%)
**17.339**	Tetradecanoic acid	C_14_H_28_O_2_	4.99
**18.658**	Pentadecanoic acid	C_15_H_30_O_2_	0.49
**19.819**	9-Hexadecenoic acid	C_16_H_30_O_2_	2.57
**19.940**	Hexadecenoic acid	C_16_H_30_O_2_	0.38
**20.126**	n-Hexadecanoic acid	C_16_H_32_O_2_	17.17
**22.775**	9-Octadecenoic acid	C_18_H_34_O_2_	3.06
**25.321**	Arachidonic acid	C_20_H_32_O_2_	0.76
**25.412**	cis-5,8,11,14,17-Eicosapentaenoic acid	C_20_H_30_O_2_	1.47
**28.936**	1,2-Benzenedicarboxylic acid, dioctyl ester	C_24_H_38_O_4_	11.93
**29.079**	Erucic acid	C_22_H_42_O_2_	0.84
**32.078**	1,3-Benzenedicarboxylic acid, dioctyl ester	C_24_H_38_O_4_	0.77
**37.431**	Cholest-5-en-3-ol (3.beta.)	C_27_H_46_O	36.71
